# Resonant Exciton Scattering Reveals Raman Forbidden Phonon Modes in Layered GeS

**DOI:** 10.1021/acs.jpclett.3c00783

**Published:** 2023-04-21

**Authors:** Joanna Jadczak, Janusz Andrzejewski, Joerg Debus, Ching-Hwa Ho, Leszek Bryja

**Affiliations:** † Department of Experimental Physics, Wroclaw University of Science and Technology, 50-370 Wroclaw, Poland; ‡ Department of Physics, TU Dortmund University, 44227 Dortmund, Germany; § Graduate Institute of Applied Science and Technology, National Taiwan University of Science and Technology, Taipei, 106, Taiwan

## Abstract

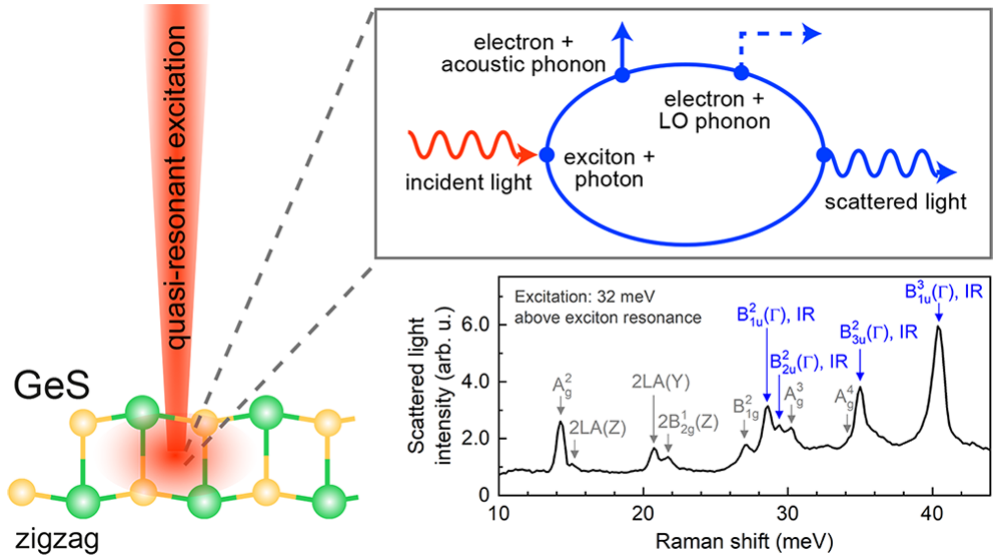

Germanium monosulfide with an anisotropic puckered crystalline structure has recently attracted much attention due to its unique optical and electronic properties; however, exciton–phonon interactions were only superficially elucidated. We study the resonant Raman scattering and the photoluminescence of the optically active Γ-exciton in layered GeS flakes and evaluate the exciton and phonon responses on variations in the excitation energy, laser-light and emission polarizations, temperature, and laser power. A double-resonance mechanism allows for observing Raman forbidden (dark) first- and second-order longitudinal-optical phonon modes whose symmetries and energies are moreover calculated by density functional perturbation theory. For (quasi)-resonant exciton excitation, the selection rules become relaxed so that a fourth-order Fröhlich intraband process is mediated by the scattering of the electron with a longitudinal-optical and an acoustic phonon. Our results demonstrate considerable coupling between phonons and photogenerated carriers in GeS flakes and the high efficiency of multiorder scattering in optical processes.

The discovery of graphene^1^ has triggered intensive studies of layered materials due to their unique physical properties in their monolayer, few-layer and bulk forms, and promising applications in new-generation devices.^2−12^ In addition to high-symmetry hexagonal-layered materials such as graphite,^1^ transition metal dichalcogenides (TMDCs)^2−6^ and boron nitride,^7^ black phosphorus (BP)^8,9^ and group-IV monochalcogenides such as GeS^10−17^ have attracted much attention owing to their anisotropic physical properties arising from a puckered single-layer structure with characteristic armchair and zigzag crystallographic directions. In contrast to TMDCs exhibiting a strong emission only as monolayers, the orthorhombic germanium monosulfide has a strong emission, both in the bulk and in the few-layer forms.^10,11^ Bulk and few-layer GeS possesses an excitonic energy gap located at the Γ-point of the Brillouin zone, with energies of about 1.6 to 1.77 eV,^10−12^ while monolayer GeS is an indirect semiconductor with a band gap approaching 2.34 eV.^13^ Due to unique physical properties such as strongly polarized absorption,^14,15^ emission,^10^ conductivity,^16^ and piezoelectricity,^17^ as well as a high ratio of external quantum efficiency and detectivity, GeS is a very promising material for applications in novel optoelectronic devices.^18,19^ These features are attributed to the peculiar energy structures of the electrons and phonons in GeS. Therefore, the comprehensive understanding of the unique physical properties of GeS calls for thorough studies of the electron and phonon structures and carrier–phonon coupling in this material.

Raman spectroscopy is an efficient tool in characterizing lattice vibrations in solids. The application of resonant Raman scattering (RRS), with a tunable excitation energy, when incoming and/or outgoing photons are in resonance with electronic transitions, allows for deep insights into carrier-phonon interactions.^20−23^ RRS is particularly efficient for studying, e.g., electron–phonon coupling, spin interactions, and nuclei effects, as well as it provides information about the exciton fine structure of low-dimensional semiconductor materials.^24,25^ RRS was also successfully used in the study of two-dimensional materials including the layered crystals of hexagonal graphene, boron nitride, and TMDCs, as well as anisotropic BP.^21−23^ Moreover, Raman forbidden phonon modes were detected in RRS spectra due to a breakdown of selection rules.^20−23^

Here, we report on the investigation of polarization-resolved RRS on GeS flakes in the range from 90 to 720 cm^–1^. Complementary photoluminescence (PL) and reflectance contrast (RC) experiments determine the energy and polarization of the neutral exciton (X) at the Γ-point of the Brillouin zone. In nonresonant Raman spectra, in agreement with previous reports,^11,26^ four Raman active modes A_g_^2^, A_g_^3^, A_g_^4^, and B_1g_^2^ are observed; however, when the excitation energy is tuned toward the X energy, 18 Raman peaks are resolved, among which 14 have not been reported previously in the backscattering geometry. The phonon modes with the symmetries B_1u_^2^, B_2u_^2^, B_3u_^2^, and B_1u_^3^, which are Raman forbidden (dark), are also observed in the optical spectra. Due to a double-resonance scattering mechanism, for the quasi-resonant excitation of the Γ-exciton, the selection rules become relaxed. By analyzing the phonon-mode dependences on variations in the excitation energy, laser-light, and emission polarizations, temperature, and laser power, we figure out that a fourth-order Fröhlich interaction plays the major role in the exciton–phonon scattering process. The corresponding Raman lines are only observed for incident and scattered photons copolarized along the armchair direction of GeS. Their line widths are nondispersive, their intensities are not suppressed by photocarriers, but they are quenched by increasing temperature. Moreover, Fröhlich coupling constants of about 0.3 indicate that spatially extended exciton-polarons are involved in the scattering process.

Bulk GeS is a layered material crystallizing in a distorted orthorhombic structure (space group D_2h_^16^) with eight atoms per unit cell; see Figure 1a–c. The lattice constants are experimentally determined to be *a* = 4.30, *b* = 3.64, and *c* = 10.47 Å,^27^ in agreement with ab initio calculations of fully relaxed lattice constants.^28^ Each Ge atom is bonded to three S atoms, and each atomic layer stack along the *c* axis as well as the unit cell contains two adjacent double layers. The puckered lattice of the layered GeS possesses an anisotropic crystal structure with two distinct orthogonal directions: An armchair atomic chain prolongs along the *a* axis (Figure 1b) and a zigzag-type connection is formed along the *b* axis (Figure 1c).

**Figure 1:**
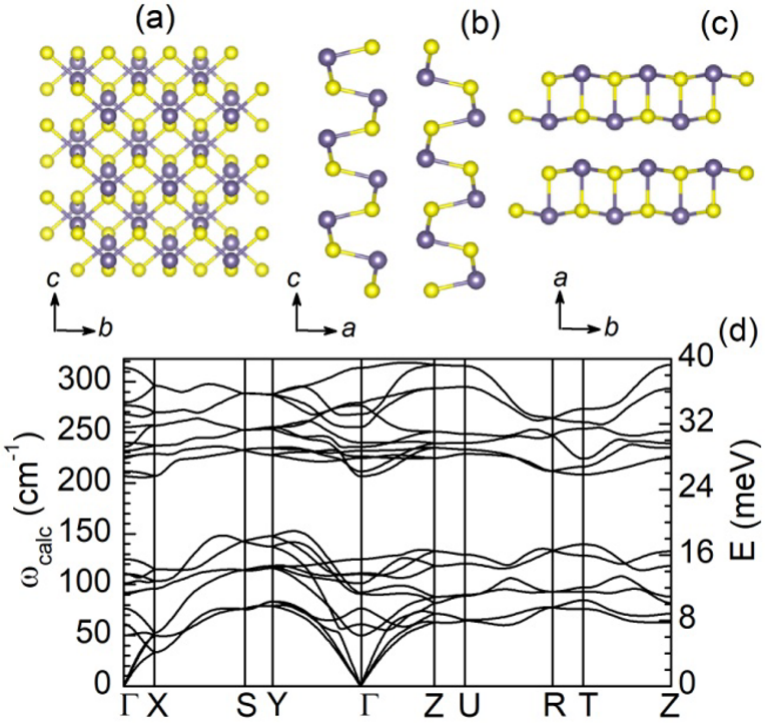
(a) Top view of the crystal structure of orthorhombic GeS. Side views of GeS: (b) armchair and (c) zigzag direction along the *a* and *b* axes, respectively. The Ge (S) atoms are depicted in the gray (yellow) color. (d) Calculated phonon dispersion of bulk orthorhombic GeS.

The unit cell with its eight atoms results in 24 branches of the vibrational spectrum. According to the group theory analysis, it has 24 irreducible zone-center phonon modes denoted by Γ = 4A_g_ + 2A_u_ + 2B_1g_ + 4B_1u_ + 4B_2g_ + 2B_2u_ + 2B_3g_ + 4B_3u_. They consist of 21 optical modes, two of them are inactive, 12 are Raman active, and seven are infrared (IR) active. The Raman active modes are 4A_g_, 2B_1g_, 4B_2g_, and 2B_3g_, whereas the IR active modes are 3B_1u_, B_2u_, and 3B_3u_. The three acoustic modes are described by the irreproducible representations B_1u_, B_2u_, and B_3u_. In the backscattering Raman geometry, the six modes 4A_g_ and 2B_1g_ (4B_2g_ and 2B_3g_) are detected when the laser light propagates along (perpendicular to) the *c* axis of the GeS crystal.^11,26,29^ The frequencies ω_calc_ and energies ℏω_calc_ of the phonon modes at the high symmetry points of the Brillouin zone are presented in Table 1, whereby ℏ is the reduced Planck constant. Moreover, in Figure 1d, the numerically calculated phonon dispersion of bulk GeS is presented. For that purpose, the ab initio plane-wave density functional theory implemented in the QUANTUM ESPRESSO code^30^ was used with a nonlocal van der Waals density functional (vdw-DF3-opt1).^31^ The wave function (kinetic energy) and density cut-offs were set to ∼1020 eV (75 Ry) and ∼8200 eV (600 Ry), respectively. The Monkhorst–Pack scheme of 4 × 11 × 10, for the k-sampling grid, was chosen. Self-consistent calculations were performed with an energy convergence criterion of ∼1.36 × 10^–7^ eV (1 × 10^–8^ Ry) and, for the relaxation of atomic positions to their equilibrium, with a force convergence criterion of ∼1.3 × 10^–3^ eV/Å (5 × 10^–5^ Ry/bohr). Using density functional perturbation theory, the dynamical matrices were established on a 3 × 5 × 5 regular mesh q-grid. Based on these matrices, interatomic force constants (IFC) in real space were calculated. The phonon dispersion shown in Figure 1d finally followed from the IFC and the Phonopy code.^32^

**Table 1:** Symmetry and Intensity Assignments of the Phonon Modes Observed in the Experiment, With Their Frequencies Derived from the Experiment, Calculation, And Literature^a^

notation	intensity	symmetry	ω_exp_ (cm^–1^)	ω_calc_ (cm^–1^)	ℏω_calc_ (meV)	ω_lit_ (cm^–1^)
p_1_	weak	2LA(X)	105	105	13.0	
	strong	A_g_^2^(Γ)	116	111.8	13.9	116,^29^ 116^11^
p_2_	weak	2LA(Z)	122	125	15.5	
g_1_	strong	2LA(Y)	167	2 × 83 = 166	20.6	
g_2_	medium	2B_2g_^1^(Z)	175	2 × 88 = 176	21.8	
	medium	B_1g_^2^(Γ)	218	215	26.7	219,^29^ 218^11^
p_3_	strong	B_1u_^2^(Γ), IR	231 (27 meV)	232	28.8	
p_4_	weak	B_2u_^2^(Γ), IR	237 (31 meV)	239	29.6	
	weak	A_g_^3^(Γ)	243	246	30.5	244,^29^ 244^11^
	very weak	A_g_^4^(Γ)	276	276	34.2	276,^29^ 277^11^
p_5_	strong	B_3u_^2^(Γ), IR	282 (33 meV)	285	35.3	275^26^
p_6_	strong	B_1u_^3^(Γ), IR	327 (41 meV)	329	40.8	325^26^
d_1_	strong	B_1u_^3^(Γ) + A_g_^1^(Γ)	378			
			327 + 52 = 379			
d_2_	strong	A_g_^2^(Γ) + B_1u_^3^(Γ)	440			
			116 + 327 = 443			
d_3_	weak	B_1g_^2^(Γ) + B_3u_^2^(Γ)	500			
			218 + 282 = 500			
d_4_	medium	B_1u_^3^(Γ) + B_1u_^2^(Γ)	558			
			327 + 231 = 558			
d_5_	medium	B_3u_^2^(Γ) + B_1u_^3^(Γ)	615			
			282 + 327 = 609			
d_6_	medium	2 × B_1u_^3^(Γ)	654			
			2 × 327 = 654			

a The energetic distances of the resonance profile maxima from the exciton resonance are listed in the fourth column, values in brackets, for the Raman forbidden modes p_3_, p_4_, p_5_, and p_6_.

We attribute the phonon modes to two groups: phonon modes at non-Γ-symmetry points as well as Raman active and Raman forbidden phonon modes at the Γ-point with frequencies below 350 cm^–1^ are assigned to group one. The second-order scattering processes at the Γ-point including Raman and IR active phonons with frequencies above 350 cm^–1^ constitute the second group. Let us start to describe the phonon modes of the first group.

The phonon modes p_1_ and p_2_ with ω_calc_ = 105 and 125 cm^–1^ are attributed to second-order scattering of longitudinal acoustic phonons 2LA(X) and 2LA(Z), respectively. Further second-order processes labeled by g_1_ and g_2_ have the frequencies 166 and 176 cm^–1^, respectively. They are positioned in the phonon frequency gap, compare Figure 1d, and are assigned to 2LA(Y) and 2B_2g_^1^(Z), respectively. The symmetry assignments for these second-order modes are consistent with the crystal momentum conservation principle. At higher frequencies the infrared active modes B_1u_^2^(Γ) at 232 cm^–1^ and B_2u_^2^(Γ) at 239 cm^–1^ are labeled by p_3_ and p_4_, respectively. In infrared reflectivity experiments performed at room temperature, the latter mode has been observed in bulk GeS.^26^ The modes p_5_ at 285 cm^–1^ and p_6_ at 329 cm^–1^ are infrared active and have the symmetries B_3u_^2^(Γ) and B_1u_^3^(Γ), respectively. Similar infrared active modes were identified in BP, which is also characterized by a puckered crystal structure.^23^

The Raman lines in the second group stem from second-order scattering processes that are realized by superpositions of intensive first-order phonon modes from the Γ-point reaching frequencies above 350 cm^–1^. We derive ω_calc_ = 378 cm^–1^ (d_1_) for the combined mode B_1u_^3^(Γ) + A_g_^1^(Γ), and 440 cm^–1^ (d_2_) for A_g_^2^(Γ) + B_1u_^3^(Γ). In both cases, Raman and IR active phonon modes determine the scattering process. Furthermore, we find at 500 cm^–1^ the d_3_ mode with the symmetry assignment B_1g_^2^(Γ) + B_3u_^2^(Γ), at 558 cm^–1^ d_4_ with B_1u_^3^(Γ) + B_1u_^2^(Γ), and at 615 cm^–1^ d_5_ with B_3u_^2^(Γ) + B_1u_^3^(Γ). The superposition of two phonons, each with symmetry B_1u_^3^(Γ), has a frequency of 654 cm^–1^ (d_6_).

In the following we focus on the PL and RC as well as Raman scattering for non- and quasi-resonant excitation of the bright exciton in a layered GeS flake. The PL and RC spectra measured at 7 K are demonstrated in Figure 2a. The PL spectrum was excited nonresonantly at 2.330 eV. The laser light propagated along the *c* axis of the GeS flake (laser light wave vector ***k***_exc_ is parallel to ***c***), while its linear polarization (electric field vector ***ϵ***_exc_) was parallel to the *a* axis. The PL exhibits five significant lines originating from the exciton (X) and most probably localized (impurity or defect) states^11^ denoted by L_1_, L_2_, L_3_, and L_4_ in the low-energy part of the PL spectrum. By comparison, in the RC spectrum only a single resonance is observed, whose energy coincides with the maximum of the X PL line positioned at about *E*_X_ = 1.776 eV. The full width at half-maximum (fwhm) of the X emission is about 15 meV. Recent works have confirmed that the X feature in the PL and RC spectra has the same origin related to a direct transition at the Γ-point of the Brillouin zone.^10,11,14^ For GeS flakes having a thickness of several tens of nm, the band gap switches from indirect (characteristic for bulk and monolayer GeS) to the direct type.^10^ The resonance in the RC spectrum and the nonzero PL efficiency underline the presence of bright Γ-excitons in our GeS flakes.

**Figure 2:**
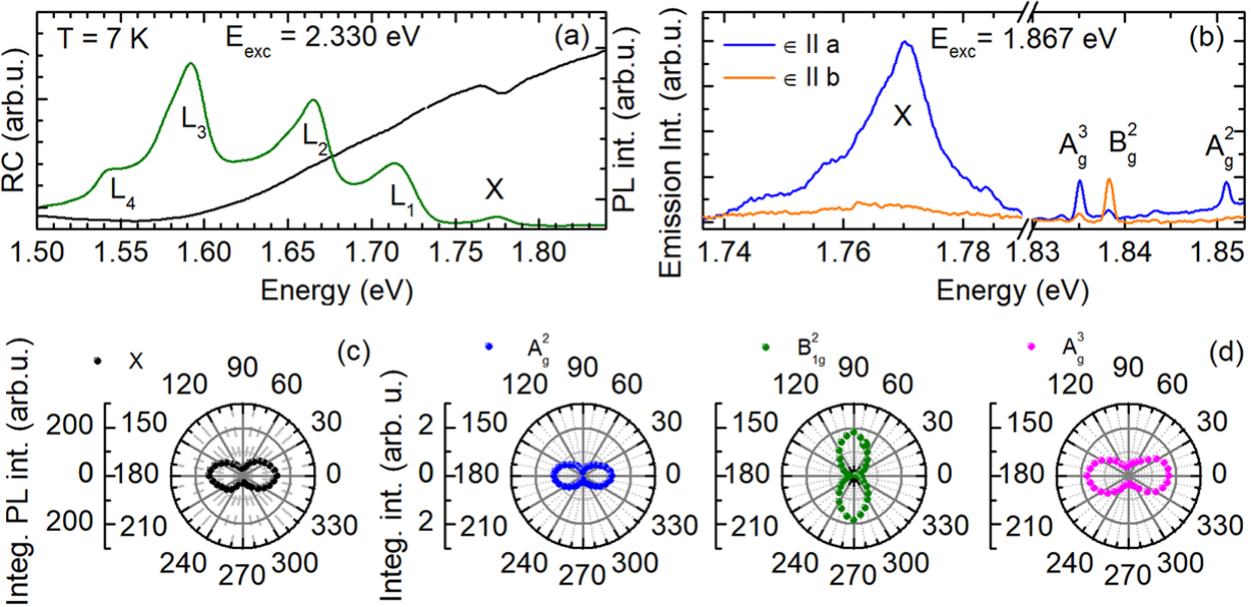
Photoluminescence, reflectivity, and Raman scattering spectra of a GeS flake as well as angular dependences. (a) Comparison between PL and RC spectra measured at *T* = 7 K and *E*_exc_ = 2.330 eV. (b) Polarization-resolved emission spectra, for 1.867 eV laser excitation and **ϵ**_exc_||***a***. Polar plots of the polarized intensities of the (c) X PL line and (d) Raman lines related to the A_g_^2^, B_1g_^2^, and A_g_^3^ modes. The angular dependences of the Raman line intensities were measured at 1.795 eV laser excitation.

Characteristic polarization properties of the X PL as well as of Raman active phonon modes are depicted in Figure 2b for quasi-resonant exciton excitation at 1.867 eV. As is clearly seen, the X emission is polarized along the armchair direction of the GeS crystal (**ϵ**||***a***), while it is suppressed along the zigzag direction (**ϵ**||***b***). The angular dependence of the polarized exciton PL intensity *I*_X_^PL^(ϕ) is presented in Figure 2c, where the rotation angle ϕ = 0° (ϕ = 90°) corresponds to **ϵ**||***a*** (**ϵ**||***b***). This angular behavior may be described by Malus law *I*_X_^PL^(ϕ) ∝ cos^2^ϕ.^10^ This spatial anisotropy of the exciton emission implies the presence of transition dipole moments aligned only along the armchair direction. Indeed, along the *a* axis the Ge and S atoms are closely spaced, giving rise to a polar bonding and, within this electric field, to an alignment of the excitonic carriers so that their dipole moment is parallel to ***a***. Thus, the measurement of the X emission polarization allows for determining the armchair and zigzag crystallographic directions.

In Figure 2b, the Raman active phonon modes A_g_^2^, B_1g_^2^, and A_g_^3^ are observed in the Stokes range from 1.853 to 1.830 eV. Considering the spectral positions *E*_ph_ of the Raman lines with respect to the laser excitation energy *E*_exc_, the phonon energies ℏω_exp_ = *E*_exc_ – *E*_ph_ correspond to our theoretical calculation and to previous reports:^11,29^ 116 cm^–1^ ≙ 14.4 meV (A_g_^2^), 218 cm^–1^ ≙ 27.0 meV (B_1g_^2^), and 243 cm^–1^ ≙ 30.1 meV (A_g_^3^). They also exhibit distinct polarization properties. The A_g_^2^ and A_g_^3^ modes are detected in the **ϵ**||***a*** polarization configuration, while the B_1g_^2^ peak is only allowed for **ϵ**||***b***. The respective angular dependences are shown in Figure 2d. They indicate that the polarization axes of the A_g_^2^ and A_g_^3^ phonon modes are oriented along the armchair direction like the polarized X emission. In contrast to this, the polarization axis of the B_1g_^2^ mode is tilted by 90° so that it is parallel to the zigzag crystallographic direction; its intensity is proportional to sin^2^ ϕ.

In what follows, we study the detection of Raman forbidden (dark) phonon modes for resonantly exciting the Γ-exciton. Figure 3a shows the Raman spectra as a function of the excitation energy. The Stokes scattering spectra were measured at 7 K in the range from Δ*E* = *E*_exc_ – *E* = 11–89 meV and the incident laser light (propagating along the *c* axis) was polarized along the armchair direction of the GeS flake (**ϵ**_exc_||***a***). The excitation energy was tuned, on the one hand, from 1.874 to 1.795 eV, corresponding to energies from 98 to 19 meV above the neutral exciton X, as indicated in Figure 3. Due to the limited emission range of the laser source (DCM-based dye laser), the exciton resonance at 1.776 eV could not be addressed directly. Nevertheless, we excited the GeS flake, on the other hand, at 1.736 and 1.748 eV, i.e., 40 and 28 meV below the exciton resonance, respectively. At these quasi-resonant excitation conditions, we are able to identify in the Raman scattering spectra 18 lines, among which 14 lines have not been observed hitherto. They are labeled by p_1_–p_6_, g_1_, g_2_, and d_1_–d_6_. Their experimentally evaluated frequencies, ω_exp_, are given in Table 1. In comparison with the theoretically assigned phonon modes, the observed lines can be attributed to acoustic phonon modes at non-Γ symmetry points (p_1_, p_2_, g_1_), IR active (Raman forbidden) phonon modes (p_3_, p_4_, p_5_, and p_6_), and second-order scattering processes, including Raman and IR active phonons (g_2_, d_1_–d_6_).

**Figure 3:**
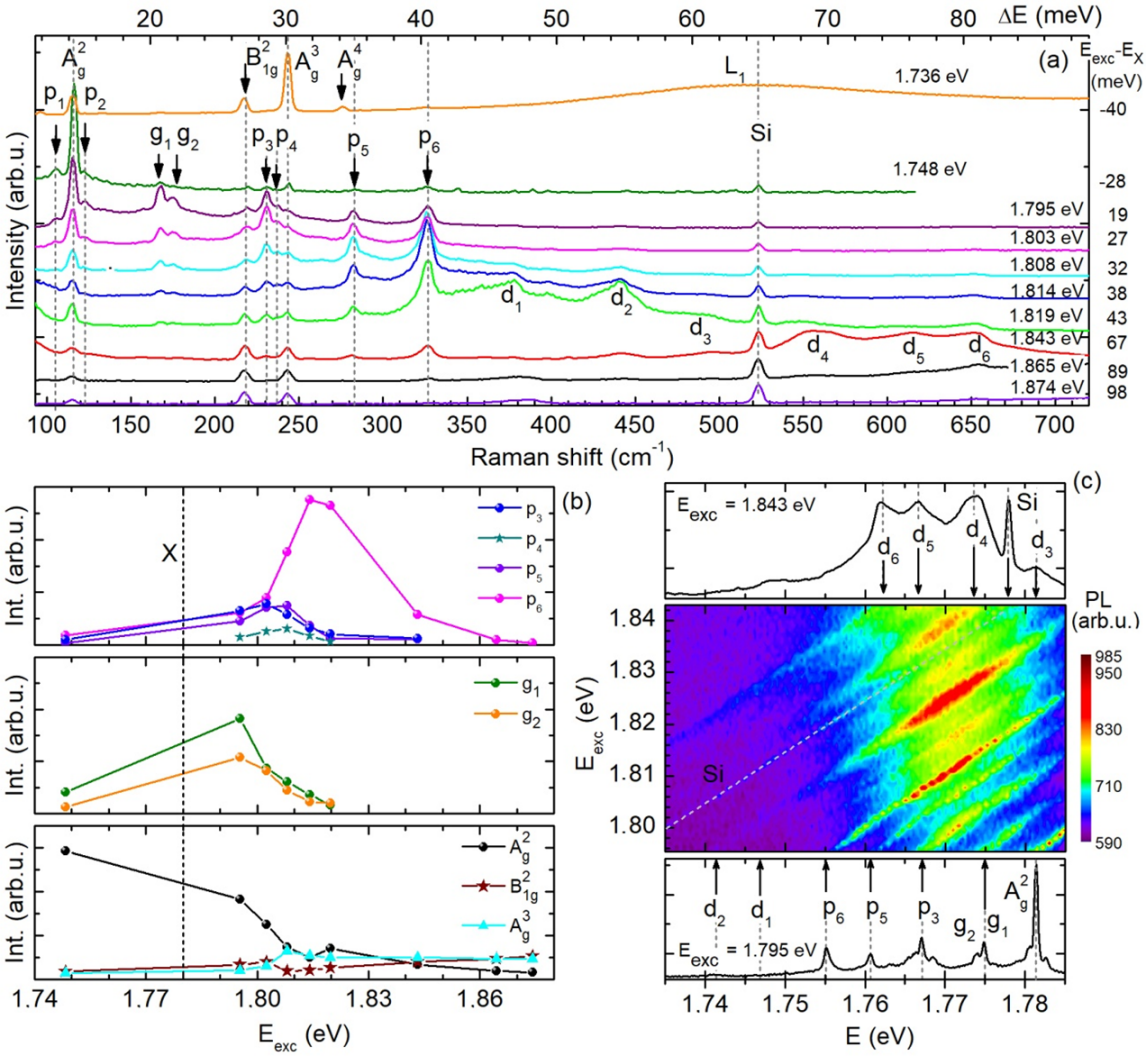
(a) Raman scattering spectra of the GeS flake for different excitation energies tuned across the X transition energy; *T* = 7 K, (**ϵ**_exc_||***a***). The vibrational mode A_g_^4^, which is also Raman active in the backscattering geometry, is detected at ω_exp_ = 276 cm^–1^, for *E*_exc_ = 1.736 eV. The Raman line at about 520 cm^–1^ belongs to the Si substrate. (b) Resonance profiles of phonon modes reveal the resonance condition for the incident photons. (c) X PL enhancement (color plot) indicates the outgoing resonance after the exciton–phonon scattering. The Raman spectra depicted in the upper and lower panels are obtained at the highest and lowest applied excitation energy equal to 1.843 and 1.795 eV, respectively.

Interestingly, the intensities of, in particular, the Raman forbidden lines are significantly enhanced. This resonant behavior is also outlined in Figure 3b which contains the resonance profiles of the different Raman lines, namely the intensities of the Raman lines as a function of the excitation energy. The Raman forbidden lines become strongly intensified at about *E*_exc_ = 1.803 eV (p_3_), 1.807 eV (p_4_), 1.809 eV (p_5_), and 1.817 eV (p_6_). The absolute error in determining the maxima amounts to ±3 meV. Comparing these values with the Γ-exciton energy *E*_X_ and the phonon energies listed in Table 1, it becomes clear that the intensities of the Raman forbidden lines are intensified when the excitation energy *E*_exc_ is equal to *E*_X_ + ℏω_exp_. Additionally, energetically scanning the Raman lines through the exciton resonance leads to a drastic increase in the exciton emission, as shown in Figure 3c. Accordingly, both the incident as well as scattered photons are in resonance with states in which the exciton is involved (double resonance). This is also the case for the second-order phonon modes d_1_–d_6_. The peaks g_1_ (2nd order acoustic phonons at Y-point) and g_2_ (2nd order optical phonons at Z-point) also seem to be enhanced in their intensities; it is not a definite observation due to the limited number of excitation energies. In contrast to that, the Raman active phonon modes at the Γ-point, e.g., B_1g_^2^ and A_g_^3^, do not significantly increase in intensity. The Raman scattering lines all have in common that their spectral positions *E*_ph_ and line widths remain constant.

A further common feature of the phonon modes p_1_–p_6_, as well as g_1_ and g_2_, is their optical anisotropy. As depicted in Figure 4, their integrated Raman intensities are at a maximum when the electric field vector of the scattered light is oriented along the armchair crystallographic direction (**ϵ**||***a***), while the intensities become negligibly small for **ϵ**||***b***. Hereby, the polarization of the incident light was polarized along the *a* axis. This angular dependence agrees with that of the X emission.

**Figure 4:**
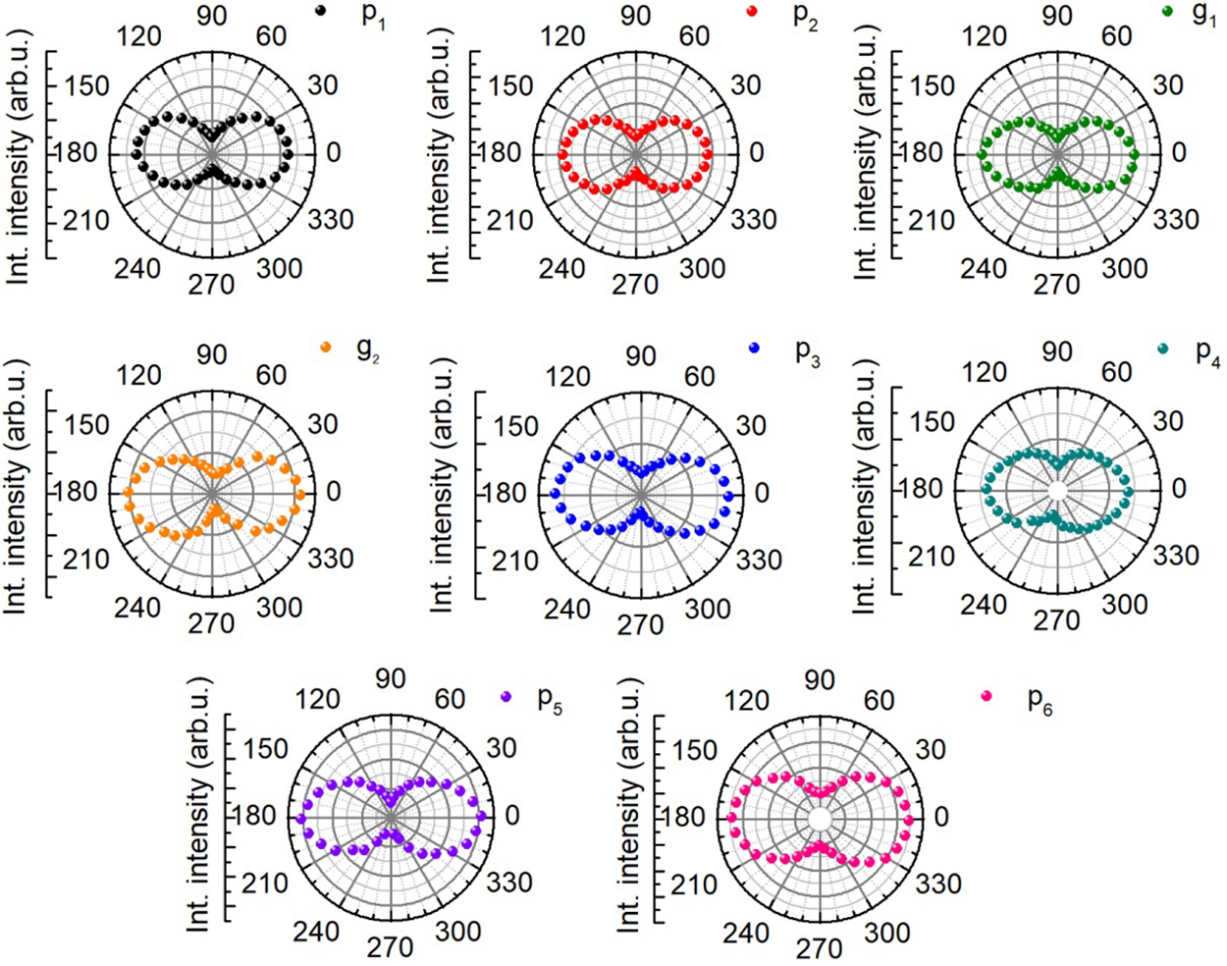
Angular dependences of the Raman line intensities p_1_–p_6_, as well as g_1_ and g_2_, measured at 7 K, and an excitation energy of 1.795 eV (**ϵ**_exc_||***a***).

We moreover study the temperature dependence of, in particular, the Raman lines with frequencies below 350 cm^–1^. Changing the temperature from 7 to 100 K yields the Raman spectra depicted in Figure 5a. The intensities of both the Raman active as well as Raman forbidden lines decrease with increasing temperature. For obtaining details about their thermal behavior, their integral intensities *I*_ph_ are shown as a function of the inverse temperature 1/*T* in Figure 5b. These Arrhenius plots allow for determining the deactivation energies of the scattering processes. Accordingly, each intensity dependence is fitted by *I*_ph_ ∝ exp(−*E*_d_/*k*_B_*T*) with the thermal deactivation energy *E*_d_ and the Boltzmann constant *k*_B_. The Raman forbidden lines p_5_ and p_6_ possess the highest deactivation energies of about (5.5 ± 1.0) meV, while the phonon lines g_1_ and g_2_ (at *Y*- and *Z*-symmetry points) and A_g_^2^ (at Γ-point, but Raman active) are quenched at lower thermal energies. For instance, the g_1_ mode has the smallest thermal deactivation energy of about 1.2 meV. The Arrhenius plots are reproducible for different GeS flakes; the values of *E*_d_ vary slightly (±0.5 meV), which may be related to the efficiency and spectral dispersion of the X emission. Furthermore, the exciton energy is red-shifted when the temperature is increased; see reflectivity spectra in Figure 5c. On average, the X energy is thermally decreased by 1.8 meV per 10 K for temperatures varying from 10 to 120 K. Thus, the difference between *E*_X_ and *E*_exc_ is not constant, but it becomes enhanced. Consequently, *E*_exc_ set at 1.795 eV meets different excitation conditions at low *T* (quasi-resonance) and high *T* (weak quasi-resonance). This thermally induced shift out of the resonance leads to an additional shrinkage of the Raman line intensities and in turn to smaller values of *E*_d_.

**Figure 5:**
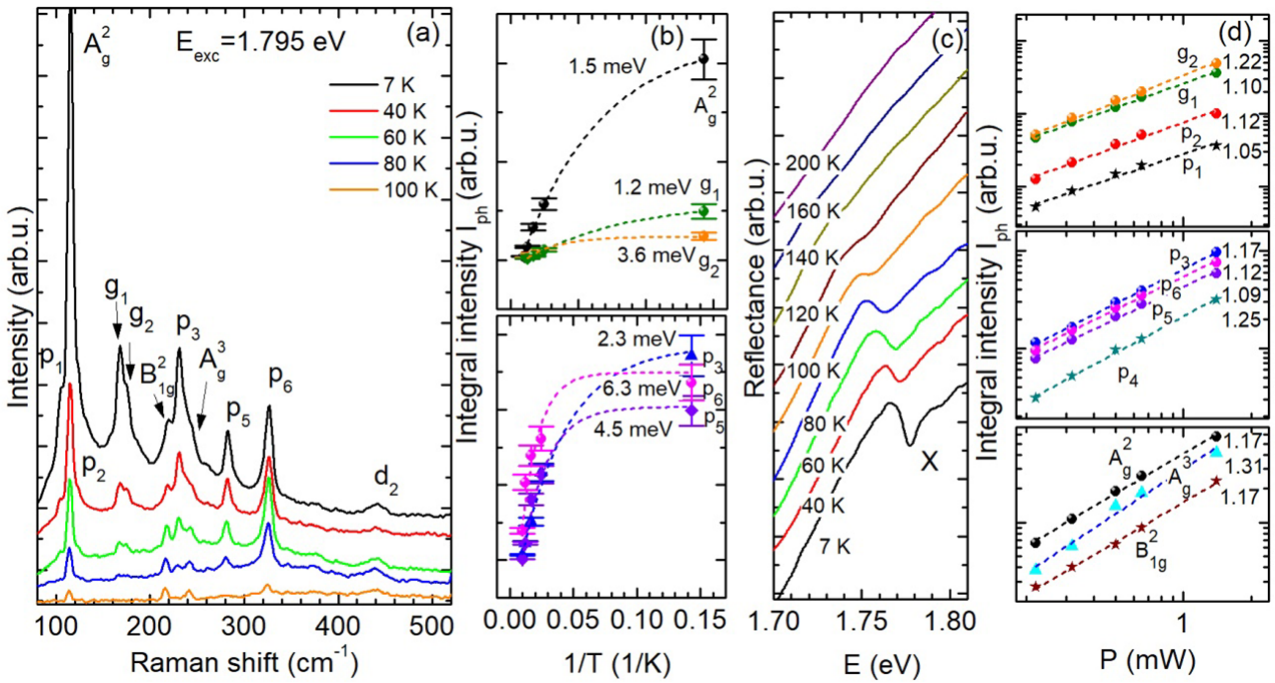
(a) Temperature-dependent evolution of Raman spectra for the GeS flake at 1.795 eV excitation (**ϵ**_exc_||***a***). (b) Arrhenius plots for different Raman lines, the exponential fits (dashes curves) yield the deactivation energies of the scattering processes. (c) Temperature dependence of reflectivity spectra demonstrating the thermally induced decrease in the X resonance energy. (d) Integral intensities of selected Raman modes as a function of the laser power; *T* = 7 K, *E*_exc_ = 1.798 eV. The dashed lines indicate the fitting curves ∝ *P*^β^. The exponent β = 1.15 ± 0.15 describes a slightly superlinear behavior.

Striking features of the IR active Γ-point phonon modes observed in the Raman spectra are that (i) their intensities are enhanced for (quasi)-resonantly exciting the Γ-exciton, in particular their resonance profiles are peaked at *E*_X_ + ℏω_exp_ (incoming resonance), (ii) they result in an enhanced X PL, indicating also an outgoing resonance, (iii) the Raman forbidden phonon modes are only detected for copolarized incident and scattered photons (**ϵ**_exc_||**ϵ**||***a***), (iv) the line widths do not show any dispersive behavior with changing excitation energy, (v) the temperature dependences yield deactivation energies of about 5.5 meV for the IR active phonon modes, while the Raman active and non-Γ-point phonons are drastically quenched by increasing temperature, and (vi) the intensities of the phonon lines increase practically linearly with increasing laser power, see Figure 5d. These properties of the Raman forbidden phonon modes observed at practically resonantly addressing the exciton in GeS flakes will allow us to evaluate the scattering mechanism.

In nonresonant Raman scattering, only Raman active phonons are detected, while IR active phonon modes remain optically nonaccessible (dark). It is based on the parity selection rule. Activating the IR active phonon modes for Raman scattering requires a nonzero transition dipole momentum. This criterion related to a breakdown of the parity selection rule and, in turn, the optical observation of the inelastic scattering by IR active phonons are realized when the incident photon energy is close to an electronic transition and the incident (**ϵ**_exc_) and scattered (**ϵ**) photon polarizations are parallel to each other.^33^ This scattering does not follow the selection rules imposed on the Raman tensor by the symmetry of the Γ-point phonons. Different microscopic mechanisms for such a selection rule relaxation have been proposed and will be discussed in the following considering our results for the GeS flakes.

When the scattering volume is in proximity to the sample surface, electric fields due to band bending may enable forbidden optical phonon scattering.^34^ In our experiments, the intensities of all phonon lines depend practically linearly on the laser power whose increase mainly corresponds to an enhancement in the number of photogenerated carriers. Their presence would alter surficial electric fields, if they were present, or they would even screen them partially so that a strongly nonlinear power dependence would be expected.^35,36^ Alternatively, considering an electron–phonon interaction based on the Fröhlich mechanism, which is inversely proportional to the dielectric constant, an increase in the carrier concentration would suppress the Fröhlich interaction and, in turn, the phonon line intensities with increasing laser power. This is not the case in our experiments.

Another possible mechanism (for nonzero matrix elements of the Fröhlich interaction) is contributed by extrinsic scattering of an exciton bound to an impurity (being the intermediate scattering state), which does not impose restrictions to the scattering wave vector ***q***, so that it is independent of the scattering geometry. In this case, the maxima of the resonance profiles would be shifted to energies lying below the resonance energy of the Γ-exciton. This energy difference would correspond to the binding energy of the impurity-bound exciton. Moreover, since the phonon momentum would be not fixed, the optical phonon lines should be dispersively broadened as a function of the excitation energy.^37^ However, the widths of the phonon lines (Raman forbidden and allowed) are practically invariant, for applying different excitation energies close to the Γ-exciton resonance.

The forbidden optical phonon scattering may arise from intraband matrix elements of the Fröhlich electron–phonon interaction in the frame of (a) a third-order process including a LO-phonon-induced intraband scattering process or (b) a fourth-order process including a scattering process of the electron with an optical and acoustic phonon, both for the Γ-exciton. In general, the longitudinal optical (LO) phonon scattering is contributed by short-range deformation potential and the long-range Fröhlich interaction.^38^ The Fröhlich interaction is induced by the electric field created by longitudinal phonons in polar materials. As the electronegativity difference between the constituents Ge and S amounts to 0.6 eV, GeS has a quite strong polarity (polar covalent bonding).^10^ Moreover, the scattering lines are copolarized; thus, we anticipate that the Fröhlich interaction dominates against the deformation potential and that the resonantly activated modes p_3_–p_6_ are LO phonons with energies ℏω_LO_ (= ℏω_exp_).

The intrinsic intraband Fröhlich interaction (a) yields a resonance profile with a maximum at *E*_X_ + ℏω_LO_/2.^39^ However, the resonance profiles of the forbidden Raman scattering lines are most intensive at about *E*_X_ + ℏω_LO_. This is actual a clear indicator that mechanism (b) plays the dominant role in our experiments on the GeS flakes.^39,40^ In BP the LO resonant Raman scattering is proposed to be mediated by an intraband Fröhlich interaction including the dark and bright excitons whose states are energetically different due to strong spin–orbit splitting.^23^ In GeS both excitons at the Γ-point are practically degenerate,^41^ so that an intraband transition–in particular with energies of a few tens of meV – between the bright and dark exciton is improbable. Thus, we conclude that the activation of the Raman forbidden phonon modes is mediated by (quasi)-resonantly exciting the Γ-exciton scattered twice by the electron-LO phonon and electron-acoustic phonon interaction. This mechanism is presented by the Feynman diagram in Figure 6a. Instead of an acoustic phonon, an impurity could be involved. Due to the slight variation in the resonance profile maxima, we propose the involvement of an acoustic phonon which shifts the profiles slightly by ℏ*s*|***q***_ac_|, where *s* is the sound velocity of the acoustic (ac) phonon. Since GeS exhibits giant piezoelectricity due to its characteristic puckered symmetry,^17^ we propose the involvement of a piezoelectric acoustic phonon in the scattering process. Taking into account a shift of ≤1 meV and a scattering vector of *q*_ac_ ≈ 1/2*a*, the sound velocity of the piezoelectric acoustic phonon in the armchair direction is approximately 1.3 × 10^3^ m/s. The large momentum transfer which occurs in the scattering event enhances the scattering cross section, despite the high order (4th) of perturbation theory involved. The large wavevector ***q***_ac_ of the acoustic phonon significantly raises the intraband Fröhlich contribution. Moreover, owing to the relaxation of the phonon wave vector it is likely that a double resonance appears, which also leads to a high Raman scattering efficiency. In our case, the incident photon resonantly excites the |X + LO⟩ state (incoming resonance), the electron of the exciton is twice scattered so that the bright exciton |X⟩ is the intermediate state whose recombination yields the scattered photon (outgoing resonance) and the system goes back into its initial vacuum state |0⟩. The respective scheme is depicted in Figure 6b.

**Figure 6:**
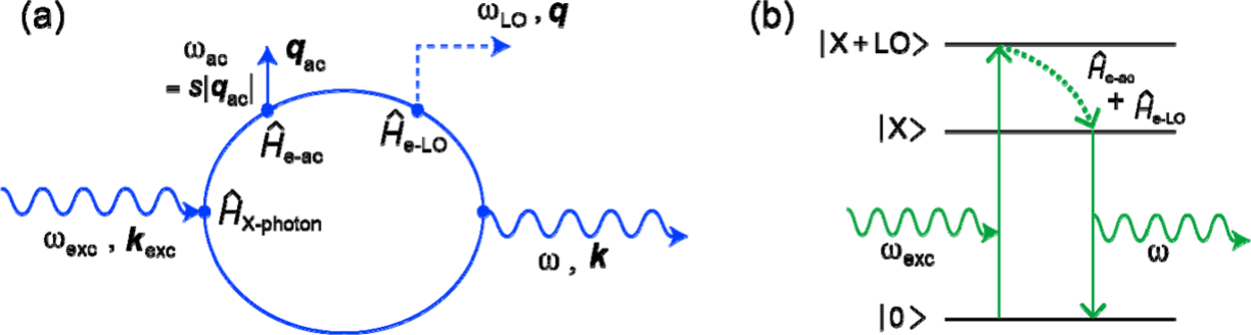
(a) Feynman diagram for Stokes scattering of a photon with initial frequency ω_exc_ and momentum ***k***_exc_ by an acoustic phonon and a LO phonon. *Ĥ*_X-photon_ denotes the exciton-photon interaction and *Ĥ*_e-ac_ (*Ĥ*_e-LO_) represents the electron-acoustic phonon (LO phonon) interaction; details can be found in ref (41). (b) Scheme for the double-resonance Stokes-scattering process in the frame of the exciton level picture.

To estimate the strength of this Fröhlich interaction we follow the approach described in ref (42). We consider the presence of Wannier excitons which is confirmed by Pastorino et al.^43^ showing that the wave function of the first bright exciton in GeS is highly delocalized and spreads over several atomic layers. The large spatial extension of the exciton is a further central property of forbidden LO scattering based on Fröhlich interaction and (quasi-)resonant exciton excitation. The scattering intensity is proportional to (*q*_LO_*a*_B_)^2^ with the exciton Bohr radius *a*_B_.^33^ Thus, forbidden phonons are observed only if the exciton Bohr radius is much larger than the lattice constant, which is the case in GeS. Accordingly, the Fröhlich coupling constant, for the electron-optical phonon interaction part, is given by
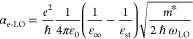
Here, *e* is the electron (e) charge, ε_0_ is the vacuum dielectric constant, ε_∞_ (ε_st_) is the optical (static) dielectric constant, and m* is the effective electron mass. For the bulk values (considering **ϵ**||***a***) ε_∞_ = 14.8, ε_st_ = 25.1,^44^m* = 0.22*m*_0_,^45^ and the phonon modes whose energies range from 20 to 40 meV, the coupling constant takes the values 0.34 to 0.24, respectively. These values lie within the relatively weak regime characteristic for the formation of large exciton-polarons.^46^ The presence of spatially extended exciton-polarons is illustrated by the comparably small deactivation energies evaluated from the temperature dependences. It is also worthwhile mentioning that the neutral exciton binding energy in GeS^41^ is significantly larger than the thermal deactivation energies. Accordingly, for quasi-resonant excitation of a large, but thermally robust exciton in GeS flakes a fourth-order scattering process is responsible for observing the actually Raman forbidden LO phonon modes p_3_, p_4_, p_5_, and p_6_ with the symmetries B_1u_^2^, B_2u_^2^, B_3u_^2^, and B_1u_^3^.

We report on resonant Raman scattering and photoluminescence of the Γ-point exciton as well as white-light reflectivity experiments in layered GeS flakes and study the inelastically scattered emission as a function of the excitation energy, laser-light and emission polarizations, temperature, and laser power. The resonant Raman scattering spectra in the range from 90 to 720 cm^–1^ exhibit 18 peaks, among which 14 have not been reported previously in the backscattering geometry. Using density functional perturbation theory and interatomic force constants in real space the phonon mode frequencies are calculated and their symmetries are defined. Due to the quasi-resonant excitation of the Γ-exciton Raman forbidden LO phonon modes with the symmetries B_1u_^2^, B_2u_^2^, B_3u_^2^, and B_1u_^3^ are observed in the optical spectra. Their intensities are enhanced for excitation energies equal to *E*_X_ + ℏω_exp_, and also the X PL demonstrates significant intensity increases caused by the double-resonance scattering mechanism. For the quasi-resonant excitation of the delocalized Γ-exciton in the GeS flakes the selection rules become relaxed so that a fourth-order process including the scattering of the electron with a LO and an acoustic phonon mediates the LO Fröhlich intraband scattering. The corresponding Raman lines are only detected for copolarized incident and scattered photons. Their line widths are nondispersive and their intensities are not suppressed by photocarriers. The thermal quenching of the Raman lines with deactivation energies of about 5.5 meV and Fröhlich coupling constants of about 0.3 indicate that large exciton-polarons participate in the scattering process. Our experiments demonstrate the relevance of exciton–phonon interactions to optical processes in two-dimensional materials and highlight that the layered GeS as direct band gap semiconductor is a material platform promising for optoelectronic applications. Moreover, our results represent a promising strategy to reveal hitherto undiscovered phonon modes using resonant spectroscopy methods.

## Materials and Methods

Layered single crystals of GeS with different areas, sizes, and thicknesses were grown by means of the chemical vapor transport method^47^ using iodine as transport agent. The crystals were prepared from their elements (Ge: 99.999% and S: 99.999%) by a reaction at 600 °C for 2 days in evacuated quartz ampules. The samples investigated by optical spectroscopy methods were 50–100 nm thick flakes that were mechanically exfoliated from bulk crystals. For this purpose, the deterministic all-dry stamping method was applied.^48^

Raman scattering experiments were performed in the backscattering geometry using a long-working distance (WD = 10 mm, NA = 0.65) 50× microscope objective for both focusing the laser light onto the flake as well as collimating the scattered light. In nonresonant Raman scattering experiments, the 633 nm (1.96 eV) line of a diode-pumped solid-state laser was used. For the resonant Raman scattering measurements the emission wavelength of a DCM-based dye laser was tuned from 670 nm (1.850 eV) to 691 nm (1.795 eV). The diameter of the laser excitation spot was about 1.5 μm. The GeS flakes were mounted on the coldfinger of a nonvibrating closed-cycle helium cryostat that allowed for varying the temperature between 7 and 300 K. The emission of the flakes was analyzed with a 0.5 m focal length spectrometer equipped with a 600 lines/mm grating and a Peltier-cooled Si-based charge-coupled device camera. The polarization was investigated by a Glan-Thompson (GT) prism combined with a λ/2 retardation plate. To eliminate the scattered laser light and phosphorescence of the dye laser, a set of short- and long-pass edge filters was used. Photoluminescence and reflectivity spectra were measured by the same experimental setup.
